# Are Patients Ready for “EARLYSTIM”? Attitudes towards Deep Brain Stimulation among Female and Male Patients with Moderately Advanced Parkinson's Disease

**DOI:** 10.1155/2017/1939831

**Published:** 2017-03-28

**Authors:** Maria Sperens, Katarina Hamberg, Gun-Marie Hariz

**Affiliations:** ^1^Department of Community Medicine and Rehabilitation, Occupational Therapy, Umeå University, 901 87 Umeå, Sweden; ^2^Department of Public Health and Clinical Medicine, Family Medicine, Umeå University, 901 87 Umeå, Sweden; ^3^Department of Pharmacology and Clinical Neuroscience, Umeå University, 90 187 Umeå, Sweden

## Abstract

*Objective*. To explore, in female and male patients with medically treated, moderately advanced Parkinson's disease (PD), their knowledge and reasoning about Deep Brain Stimulation (DBS).* Methods*. 23 patients with PD (10 women), aged 46–70, were interviewed at a mean of 8 years after diagnosis, with open-ended questions concerning their reflections and considerations about DBS. The interviews were transcribed verbatim and analysed according to the difference and similarity technique in Grounded Theory.* Results*. From the patients' narratives, the core category “Processing DBS: balancing symptoms, fears and hopes” was established. The patients were knowledgeable about DBS and expressed cautious and well considered attitudes towards its outcome but did not consider themselves ill enough to undergo DBS. They were aware of its potential side-effects. They considered DBS as the last option when oral medication is no longer sufficient. There was no difference between men and women in their reasoning and attitudes towards DBS.* Conclusion*. This study suggests that knowledge about the pros and cons of DBS exists among PD patients and that they have a cautious attitude towards DBS. Our patients did not seem to endorse an earlier implementation of DBS, and they considered that it should be the last resort when really needed.

## 1. Introduction

Deep Brain Stimulation (DBS) of mainly the subthalamic nucleus (STN) has become an established surgical procedure for patients with advanced Parkinson's disease (PD) [1–3].

Nevertheless, it is not unusual that the beneficial effect of DBS is mitigated by various side-effects such as dysarthria, decrease in verbal fluency, and changes in behaviour, fatigue, and depression [4–6]. Careful selection criteria of patients considered for DBS have been established, including Levodopa response, age, normal brain MRI, good cognition, and realistic expectations [3, 7]. Following adequate information about the pros and cons of the procedure [8], the final decision to undergo surgery will be taken by the patient.

Research on the decision-making process of patients having already undergone DBS for PD had shown in retrospect that the individual patient's knowledge about (and attitude towards) DBS had been crucial for their final decision to undergo DBS [9]. However, non-operated upon patients' own thoughts, considerations, and apprehensions concerning advanced therapy for PD have received scarce attention in the literature [10, 11]. This issue is all the more interesting in light of existing gender differences, with more men than women undergoing DBS for PD [12–15] and given the current trend of suggesting DBS earlier in the disease progress [16–18].

The aim of this qualitative study was to explore, in female and male patients with medically treated, moderately advanced PD, their knowledge, feelings, and reasoning about DBS.

## 2. Material and Methods

### 2.1. Participants

In order to enroll in this study patients who may have had reason to consider DBS as a treatment alternative, a strategic selection was used: a nurse specialized in PD at Umeå University Hospital helped us to identify patients with PD who despite high and/or frequent doses of dopaminergic medication experienced difficult symptoms and problems in daily life. There were 36 patients (23 males, 13 females) who fulfilled these criteria. Information about the study was sent to them and they were asked if they agreed to participate in an interview. One reminder was sent to those who did not answer. Twenty-one patients (14 men) accepted to be interviewed. One 80-year-old patient was excluded since he would not have been eligible for DBS due to high age. Three additional women were recruited along the same criteria after contact with Parkinson's Disease Society.  Table 1 shows the description of the 23 enrolled patients.

The local ethical board at Umeå University approved the study, and all patients gave written informed consent before the interview (D.No: 2012-36-32M).

### 2.2. Data Collection

Data were collected through qualitative interviews [19, 20]. The majority of the interviews were conducted face to face by one interviewer (MS, GMH, or KH) in a setting chosen by the patient, usually in the patient's home. Due to practical difficulties (e.g., long distances) four patients were interviewed by telephone. The interviews were semistructured, with open-ended questions concerning broad areas, such as how the patients felt and reacted when they received the diagnosis, their experiences of PD and its treatments over time, how it has been and how it is now to live with PD, their knowledge about treatments other than oral medication, especially DBS, and how they felt and thought about future treatment. In this paper, we focus on the patients' knowledge, feelings, and reasoning about DBS. Sample questions related to this focus included the following:* “Can you tell about the treatment that you currently have for Parkinson's disease?”; “Do you know of other treatments than oral medications?”; “How did you learn about these other treatments?”; *and* “What do you think and feel about DBS as a treatment for PD?”* The interviewer tried to facilitate the narrative by follow-up questions such as,* “Please, can you explain further”; “What do you mean?”; “Please, could you give me an example?”*

Each interview lasted 60–140 minutes and was digitally recorded and transcribed verbatim.

In addition to the interview, each participant completed a short questionnaire about sociodemographic information. The patients were also asked to assess the overall impact of PD on their health by answering the questions* “In general, how do you perceive your overall health on a five-point scale (excellent, very good, good, fair, bad)?”* and* “How do you experience the overall impact of your Parkinson's disease (mild, moderate, severe)?”*

The patients' Levodopa equivalent daily doses (LEDD) at time of the interview were obtained from the patients' medical record.

### 2.3. Data Analysis

#### 2.3.1. Qualitative Analysis of the Interviews

According to qualitative research design [19], preliminary analyses of the transcriptions were conducted in parallel with the interview process. The authors could thereby learn and reflect during the interview process, refine interview questions, and be alert when new aspects were described.

The main analysis of the interviews was made according to the constant comparison technique in Grounded Theory [19, 20]. The analysis contained the following phases:

1. In a first phase all researchers separately read and coded three interviews and then met to compare codes and discuss content and meaning of the participants' experiences. Case narratives summarizing the essentials of each interview were written down. Another three interviews were then coded, compared, and summarized, and this process of sorting the data continued until all interviews were worked through and summarized in case narratives.
2. In a second phase all interviews were reread by the first author and passages that concerned the participants' thoughts, reflections, and utterances about future treatment and DBS were identified, cut out, and organized in separate* “considerations-about-treatment” *files, one for each participant. These files were read and systematically coded and compared for similarities and differences by all researchers separately. In joint sessions the codes were compared and discussed, and categories and subcategories were elaborated. A core category embracing the content and meaning in the participants' narratives was also established.
3. Thereafter the “*considerations-about-treatment*” files were analysed specifically for similarities and differences between men and women.
4. Finally the whole interviews were reread to ensure that the categories and interpretations could be recontextualized into the interviews, that is, that the results were grounded in the data.

### 2.4. Statistical Analysis

Descriptive continuous variables were presented as average ± standard deviation and range by use of the SPSS for Mac 21.0. A *p* value < 0.05 was considered significant.

## 3. Results

### 3.1. Demographic Data and Clinical Outcome

Table 1 shows the sociodemographic and clinical characteristics of the participants, their self-assessed general health, and the overall impact of PD on life as a whole, as well as the Levodopa equivalent daily dose (LEDD). The mean disease duration was 7.8 years and the mean LEDD was 1186 mg. One patient was treated with Duodopa pump. Thirteen patients (4 women and 9 men) reported that they needed help in some of the daily activities. All but one patient considered that PD had a moderate overall impact on their life (Table 1).

### 3.2. Interviews

The participants displayed interest and engagement in the interview. They described in detail their symptoms and how these impacted on their everyday life. The most common symptoms reported by the patients were in various combinations: shaking, stiffness, wear-off and fluctuations, involuntary movements, cramps, fatigue, gait problems, low mood, and sensitivity to stress. There were no differences in symptom profile between men and women.

With respect to DBS, all participants were knowledgeable about it, and shared their views and reflections about DBS as a potential additional treatment. The sources of their knowledge were information from (and discussions with) medical staff, as well as information from the Internet, from watching TV-programs and by reading newsletters published by the patients' society. Several participants had also met other people who had undergone DBS for PD.

The analysis of the interviews resulted in the core category “Processing DBS: balancing symptoms, fears and hopes.” This core category was underpinned by two main categories: “*Neurosurgical treatment requires careful consideration”* and “*Timing of concurrent issues of importance for DBS.” *Each of these two categories was supported by three and four subcategories, respectively (see Table 2). In the following, the categories and subcategories are presented and illustrated with quotes from the participants. The participants are given fictitious numbers from Mr. 1 to Ms. 23.

#### 3.2.1. Processing DBS: Balancing Symptoms, Fears and Hopes

The participants' main opinion about DBS as a treatment alternative was that DBS was not on their agenda for the time being. However, most of our interviewees considered that DBS might become an alternative later due to progress of the disease or to drawbacks and inefficacy of medication. Their current situation and the degree of difficulties that they experienced in daily life, as well as their hopes for research and discoveries of new and better treatment options for PD, also impacted on the way women and men reasoned about eventual DBS treatment. 

*Neurosurgical Treatment Requires Careful Consideration*

*(1) Worries Related to the Neurosurgical Procedure*. Both men and women expressed worries about undergoing a neurosurgical intervention and the potential risk of damaging a very important and sensitive organ. Mr. 1 described his fascination about the capacity of the brain and at the same time his fear of being damaged during surgery*: “I remember a fishing tour, it is twenty-five years ago, I can spot it in a split second…”* and he continued “*they* (the electrodes)* are very close to the memory centre.”*

Some of the participants' considerations consisted of more general expressions about surgery being something that always could pose a risk, whereas other concerns were more specifically related to the surgical procedure per se, such as being attached to the surgical equipment. Such thoughts implied feelings of uneasiness, as phrased by Mr. 10, “*would you like to be strapped up?*” The participants expressed both positive and negative concerns about the new routine of having DBS under general anaesthesia: on one hand, they felt relief at being asleep during drilling of the skull, and on the other hand they expressed fear of being totally without control during the course of surgery. 

*(2) Cautious Attitudes towards Outcome after DBS*. Both men and women were concerned about what they perceived to be an inconsistent outcome after DBS. They had noticed that some friends and acquaintances who had DBS felt very well while others seemed to have deteriorated to a state worse than before surgery. Mr. 1 referred to the following observation of a friend:* “I know a person who was convinced DBS would turn out well and that was also the case initially, but then he encountered complications and now he is not that well anymore.”* The participants' thoughts and considerations were mainly related to a potential negative outcome after DBS rather than to possible positive effects, and the risk of impaired balance after DBS was frequently mentioned as a concern.

Another common perception among the interviewed patients was that after DBS some patients seemed to need higher and more frequent doses of medication. The participants regarded this as a negative outcome of DBS. Ms. 14 said,* “I think that they *(fellow people with PD after DBS)* are in need of lots of medications.”* Further, some of the participants had met people who after DBS did not seem to be their* “usual self”* any more. They were more low-spirited and nearly depressed, as told by Ms. 16, who stated,* “I must say that they became low, I would say depressed and their reasoning was in a different way, as well”; *and Mr. 3 stated, “*I think they have become more quiet, one might say a bit less positive.*” These participants implied thus that there is a possible risk that DBS may induce personality changes. 

*(3) Concerns about Suitability of DBS for One's Own Symptoms*. The participants expressed concerns about whether they themselves would be suitable candidates for DBS surgery. The interviewees whose tremor was their main symptom considered that the shaking was difficult to treat only with oral medication, and they also knew that DBS might be efficient for alleviating tremor, “*I would probably be a good candidate for DBS because I am shaking…”* (Ms. 18). On the other hand, participants with impaired balance explained that they were less likely to be ideal candidates for DBS and Mr. 7 said that his neurologist considered that* “to offer me surgery would not be a good idea because it can lead to worsening of gait and some patients may get poorer balance.”*

*Timing of Concurrent Issues of Importance for DBS*

*(1) Bringing Up the Issue of DBS*. The participants considered that they had enough knowledge about DBS as a treatment alternative, and the majority of them expressed no wish or need for more discussion about DBS for the time being.

Three women reported that they had found it difficult to consider DBS surgery when their clinician suggested it early in the course of the disease, and when one of them was referred to the DBS team, she declined to undergo the presurgical evaluation. Six of the men had been offered DBS and two of them declined to undergo presurgical screening. Among the four men who were assessed in view of DBS, two were not found to be ill enough, while the two others eventually understood that they were not considered suitable due to early signs of cognitive decline. Both of them expressed that it would have been easier to accept the denial of DBS had they received a careful explanation of the reasons, as exemplified by Mr. 1: “*yes, my understanding perhaps would have been better if I had had a proper explanation as to why they instead recommended pump to me.”*

Still, bringing up the issues about DBS was considered highly relevant for two of the male participants and they were awaiting the right moment to bring it up themselves, as Mr. 12 put it:* “well, absolutely, I can bring up DBS myself, but today I do not feel it is the right time.” *

*(2) Utilizing the Treatment Alternatives Gradually, Step by Step*. The participants considered oral medication as the basic treatment, and they were hoping to be able to keep their current treatment stable for as long as possible. They considered advanced treatment alternatives, such as infusions and injections and DBS as a limited treatment resource. These different treatment alternatives were described as something linear, to be taken step by step. The typical description was that medication was followed by an increase of oral medication, thereafter apomorphine injection pens, and then pumps and finally ending with DBS. This can be illustrated by Mr. 8 in the following:* “I have to put up with certain things because I know that the more medications I ‘waste' the more I tear on future resources”* and Ms. 19 stated that* “DBS is the last* (treatment alternative).” To utilize the last step, that is, DBS, was something unwanted, and, for the participants, it implied having nothing left to turn to if needed after surgery. Mr. 13 explained,* “So I'm still acting cowardly. You also need to have some treatment alternative left.”* Thus, the majority described DBS as a step they rather would postpone as long as possible. For a few patients though, DBS was more of a natural treatment step when medication no longer efficiently could control the symptoms of the disease, as described by Ms. 20, “*if the impact of medication ceases, then there is more *(DBS)*, like a continuum.”*

*(3) Considering Disease Progression and Life Situation*. Even if most participants did not consider DBS in their current situation, they envisaged it as an alternative later on, if, or when, the symptoms became even worse. Both women and men expressed that when the disease had progressed to a level when they would have great difficulties managing their lives, DBS might be an additional treatment option. At a certain point of disease progression, any treatment that may provide a better life could be considered. This reasoning was put forward by Ms. 23 in the following:* “When I no longer am able to brush my teeth, then I might consider DBS” *and by Mr. 6: “*I would keep away from it *(DBS)* as long as possible. But it is hard to say, if you are struck by these difficulties you might feel that you would do anything ….”*

*(4) Hoping for Future Breakthrough in PD Research*. The participants were aware of the importance of research for improved life conditions for persons with PD, and they expressed hope that research would open up for totally new treatments. Some conveyed a hope for a real cure of the disease, rather than only better or newer pills to keep the symptoms at a manageable level, as uttered by Ms. 20,* “and then some researcher will find something marvelous.”* The patients' wishes for research-driven new and better treatments were particularly focused on nonsurgical options rather than new surgical procedures. They hoped for more efficacious oral medication, for reducing the numbers of daily doses to only one intake a day and for an easier handling of equipment when using pumps. Expectations and hope related to DBS were expressed in more general terms, such as wishes for even better surgical skills and techniques. There was awareness about stem cells research and also about alternative nonmedical treatment such as dietary advice, for example, eating blueberries.

However, even if most participants hoped for breakthroughs in research they underlined that research takes time and Mr. 4 confessed that he nowadays had low expectations:* “I*'*ve been interested but I have sort of given up on that now. It takes so long before it becomes available, stem cells and so on.”*

## 4. Discussion

The aim of this interview study was to explore attitudes towards (and perceptions of) DBS, among women and men with medically treated, moderately advanced Parkinson's disease, who could have had reasons to consider DBS as a treatment option. The most interesting findings were that both women and men were quite knowledgeable about DBS but they did not feel that DBS was an option for them for the time being. They had respect for DBS as being a serious surgical procedure done on the brain, and they considered that it should be kept as a last resort. They were also aware of its side-effects such as impaired balance and personality changes. In contrast to what has been reported in the literature [21–26] and what is commonly depicted in the lay press [27], our patients kept low expectations from DBS. However, the patients were also aware of the symptom profiles that are commonly considered to benefit from DBS (such as tremor) as well as the contraindications, such as balance problems and cognitive decline. There were no differences in those respects between male and female participants. On the whole, despite having had the disease for several years and despite the myriad of symptoms that they described, there was an agreement among patients that DBS should be utilized as a last treatment, when all other options were exhausted. This approach, conveyed by the patients themselves, is at odds with the recent “EARLYSTIM” trend in the literature in favor of proposing DBS for patients earlier in the disease progression [16–18].

### 4.1. Patients' Considerations about DBS

How come that our patients showed more reserved and less enthusiastic attitudes towards DBS than what one can commonly find in the literature? [16–18]. There may be several explanations to this: our patients had in general a high level of education with 78% of them having a high school or university degrees (Table 1), which could imply that they were able to better judge information conveyed by the lay media and health care professionals. Additionally, 83% of our patients were members of a Parkinson's disease organization (Table 1) and hence may be well knowledgeable about the disease and its various treatments modalities, including their side-effects, as shown in other studies [8, 28]. Another factor to consider is that several of our patients had friends and acquaintances who had had DBS and they could thus see that the reality of DBS was sometimes different from the glamour, in particular concerning the side-effects. Patients who were on DBS may have conveyed to our participants a sense of disappointment despite the motor improvement [29]. The fact that our patients rated the impact of PD on their life as moderate (Table 1) and did not have a very long disease duration did not motivate them to consider a surgical procedure that may harbor complications and side-effects: they felt that for the time being they may have more to lose than to gain from DBS. In this respect, it is important to underline that it may be difficult for patients to admit that a chronic progressive illness is “severe” such that it may lead to seeking a treatment that they consider as a “last resort.” Hence, our patients would most probably not have submitted themselves to an “EARLYSTIM” procedure [16, 30]. Three of the 13 men and five of the 10 women who were interviewed were still professionally active and this could also be a factor that influenced their attitude to DBS especially in relation to the possible side-effects from surgery that all patients were aware of. Finally, our patients expressed hope that research would bring about other nonsurgical treatments that they would benefit from, enabling them thus to avoid a surgical procedure on their brains.

### 4.2. Gender Differences in Perceptions of DBS?

Earlier studies have shown that, in relation to the gender prevalence of PD, women are underrepresented among those treated with DBS [12, 14, 15, 31]. The reason for this is unknown but it has been suggested that women might be more “afraid” of (and hesitant towards) neurosurgery compared to men [32]. Our results here showed that the narratives and ways of reasoning about DBS were similar in men and women. Both men and women contributed to all subcategories and categories. Both expressed some worries for surgery and its risks and had modest expectations on the positive effects of DBS. Likewise, both men and women considered DBS to be a treatment modality to postpone until the symptoms were too difficult to cope with and to consider when no other treatment option was left. Consequently, our results do not give evidence for any differences in perceptions and attitudes towards DBS among men and women that could explain the male predominance among patients treated with DBS for Parkinson's disease [12, 15, 16]. Additionally, it seems that our patients may not endorse an “EARLYSTIM strategy” for treatment of their PD, such as has been advocated recently in the literature [16, 17].

## 5. Limitations of This Study

For this study, near half of the patients who were invited to participate either declined the invitation or did not reply. This may have introduced a selection bias in favor of patients who are more outgoing and willing to discuss their disease and attitudes to DBS. Additionally, our participants are all living in Sweden and the results may not be applicable elsewhere.

## 6. Conclusions

In conclusion, our study showed that patients with moderately advanced PD who would be potential candidates for DBS had indeed good knowledge about the pros and cons of this treatment modality and expressed a realistic view about its potential limitations. They were not ready yet to submit to “early” DBS; they perceived DBS as a last resort that should be carefully considered only if absolutely needed. There were no differences between men and women concerning their reasoning and attitude towards DBS.

## Figures and Tables

**Table 1:** Sociodemographic and clinical characteristics of 23 participants (10 women) with Parkinson's disease.

	Whole group	Men (%)	Women (%)	*P*
Number of Participants	23	13/(56.5)	10 (43.5)	
Age	Mean ± SD (range)	Mean ± SD (range)	Mean ± SD (range)	
Age at diagnosis	52.4 ± 7.15 (40–63)	53.7 ± 7.5 (41–63)	50.7 ± 6.7 (40–61)	ns
Years since diagnosis	7.8 ± 4.7 (1–19)	8.0 ± 4.3 (3–17)	7.6 ± 5.5 (1–19)	ns
Age at interview	60.2 ± 6.8 (46–70)	61.6 ± 7.2 (46–70)	58.3 ± 6.1 (47–67)	ns
LEDD (mg)^€^	1185.5 ± 555.4 (525–2322)^€^	1356.7 ± 618.9 (525–2322)^€^	889.6 ± 250.4 (600–1310)^€^	ns
Number of daily doses^*¥*^	5.3 ± 1.8 (3–9)	5.9 ± 1.9 (3–9)	4.3 ± 1.2 (3–6)	0.045
Number (%) of patients who needed assistance in some daily activities	13 (56.5)	9 (69.2)	4 (40)	
Civil status	*N *(%)	*N* (%)	*N* (%)	
Cohabitant/single	19 (83)/4 (17)	11 (85)/2 (14)	8 (80)/2 (20)	
Level of education	*N* (%)	*N* (%)	*N* (%)	
Primary school	5 (21.7)	3 (23.1)	2 (20.0)	
High school	7 (30.4)	4 (30.8)	3 (30.0)	
University	11 (47.8)	6 (46.2)	5 (50.0)	
Employment status at time of interview	*N* (%)	*N* (%)	*N* (%)	
Working full time	1 (4.3)	1 (7.7)	0	
Working part time & sick-leave part time	7 (30.4)	2 (15.4)	5 (50.0)	
Sick-leave full time	8 (34.8)	4 (30.8)	4 (40.0)	
Retired	7 (30.4)	6 (46.2)	1 (10.0)	
Perceived general health at time of interview^#^	*N* (%)^#^	*N* (%)^#^	*N* (%)	
Excellent	1 (4.5)	0 (0.0)	1 (10.0)	
Very good	5 (22.7)	3 (25.0)	2 (20.0)	
Good	5 (22.7)	2 (16.7)	3 (30.0)	
Fair	10 (45.5)	7 (58.3)	3 (30.0)	
Bad	1 (4.5)	0 (0.0)	1 (10.0)	
Overall impact of PD on life at time of interview	*N* (%)	*N* (%)	*N* (%)	
Mild	1 (4.3)	1 (7.7)	0	
Moderate	22 (95.7)	12 (92.3)	10 (100.0)	
Severe	0	0	0	
Number of members of PD society (%)	19 (82.6)	11 (84.6)	8 (80.0)	

L-dopa = Levodopa.

LEDD = Levodopa equivalent daily doses.

^€^Missing data in 4 (1 male) patients.

^*¥*^Missing data in 2 female patients.

^#^Missing data in 1 male patient.

**Table 2:** A core category underpinned by two main categories. Each main category is supported by three and four subcategories, respectively.

Core category	Processing DBS: balancing symptoms, fears and hopes	
Main categories	Neurosurgical treatment requires careful consideration	Timing of concurrent issues of importance for DBS

Subcategories	(1)* Worries related to the neurosurgical procedure* (2) *Cautious attitudes towards outcome after DBS* (3)* Concerns about suitability of DBS for one's own symptoms*	(1)* Bringing up the issue of DBS* (2) *Utilizing the treatment alternatives gradually, step by step* (3) *Considering disease progression * *and life situation* (4)* Hoping for future breakthrough in PD research*
